# Evaluation of Evolux™ Intraocular Lenses in Cataract Surgery: Clinical Outcomes and Patient Satisfaction

**DOI:** 10.3390/jcm13237404

**Published:** 2024-12-05

**Authors:** Antonio Cano-Ortiz, Álvaro Sánchez-Ventosa, Timoteo González-Cruces, Marta Villalba González, María Dolores López Pérez, José Carlos Díaz-Ramos, Juan J. Prados Carmona, Victor Tejerina Fernández, Daniel Elies Amat, Alberto Villarrubia

**Affiliations:** 1 Department of Anterior Segment, Cornea and Refractive Surgery, Hospital Arruzafa, 14012 Cordoba, Spain; 2 Department of Health and Biomedical Sciences, Universidad Loyola Andalucía, 41704 Sevilla, Spain; 3 Department of Ophthalmology, Reina Sofia University Hospital, 14004 Cordoba, Spain; 4 Instituto de Microcirugía Ocular (IMO), 28035 Madrid, Spain

**Keywords:** Evolux, intraocular lenses, surgery

## Abstract

**Objective:** This study aimed to evaluate the initial clinical outcomes of and patient satisfaction with the newly introduced extended monofocal Evolux™ intraocular lens following cataract surgery. **Methods:** A retrospective study was conducted, involving 18 patients (36 eyes) who received Evolux™ lenses bilaterally. The inclusion criteria comprised individuals over 40 years old with no active eye diseases, excluding cataracts, and no postoperative complications. Various parameters were evaluated, including distance, intermediate, and near visual acuity; refraction; defocus curves; dependency on spectacles; and patient satisfaction. Patient satisfaction and visual quality of life were measured using the Catquest-9SF and QOV questionnaires. **Results:** The average age of the patients was 72.7 years, with the majority being women (77.8%). The results demonstrated excellent postoperative visual acuity at different distances. The mean logMAR values for uncorrected visual acuity were −0.04 for distance vision, 0.15 for intermediate vision, and 0.35 for near vision. The defocus curve indicated good tolerance, with visual acuity exceeding 20/20 in significant defocus ranges. Most patients expressed high satisfaction with their vision without spectacles, particularly for distance and intermediate vision. The need for spectacles for near vision was notably reduced. Furthermore, there was a low incidence of photic phenomena like halos and glare, contributing to the overall high patient satisfaction. **Conclusions:** Evolux™ lenses were found to be an effective choice for cataract surgery, providing excellent visual acuity at various distances and high patient satisfaction.

## 1. Introduction

The advancement of intraocular lens (IOL) designs has played a crucial role in enhancing visual outcomes for patients undergoing cataract surgery. For many years, traditional monofocal IOLs have been the standard, offering excellent distance vision. However, they have limitations when it comes to near and intermediate vision [1]. With the increasing use of devices such as mobile phones, tablets, and computers, intermediate vision has become more crucial than ever. Traditional single-vision lenses are designed for distance vision, leading patients to require spectacles for other distances [2,3]. This reliance has emphasized the need for a change in IOL designs to better address the visual needs of an increasingly active and technologically dependent population.

One notable innovation is the extended monofocal lens, which provides enhanced intermediate vision without the traditional side effects associated with multifocal lenses, such as halos and glare [4,5,6]. Unlike multifocal IOLs, which aim to deliver clear vision at multiple distances, Monovision Plus IOLs concentrate on improving distance and intermediate vision while minimizing visual complications, offering a more natural experience for the patient. These IOLs present an appealing alternative for patients seeking to reduce their reliance on spectacles without contending with the side effects of multifocal IOLs [6,7,8,9].

The development of extended monofocal IOLs is based on an advanced optical design that slightly expands the depth of focus, providing patients with clearer vision at intermediate distances without compromising the clarity of long-distance vision. Additionally, these lenses are designed to minimize spherical aberrations, which improves overall visual quality and contrast sensitivity. This evolution in intraocular lens design represents an important advance in vision correction, especially for those patients who seek to maintain clear vision at intermediate distances without the typical side effects of multifocal lenses [10].

The Evolux™ is an extended monofocal intraocular lens that has been specifically designed to overcome the limitations of traditional monofocal IOLs. Unlike multifocal lenses, the Evolux™ IOL uses a non-diffractive optical profile that extends the depth of focus, providing clear and continuous vision from intermediate to long distances without the adverse effects of halos and glare [2,11]. This design responds to the needs of a population that requires high-quality vision for everyday tasks such as reading, working on screens, and driving, activities in which intermediate vision plays a crucial role.

The Evolux IOL™ is made from a hydrophobic material, which is associated with enhanced lens biocompatibility and a potential reduction in visual distortions under low-light conditions. Its optical design aims to minimize spherical aberrations, which may contribute to improved contrast sensitivity and visual clarity. These characteristics make it a viable option for patients requiring effective intermediate vision correction while maintaining good distance vision quality [11,12].

This study aimed to present early clinical outcomes and patient-reported experiences in the initial postoperative period with the new Evolux™ IOL. The study evaluated distance, intermediate, and near visual acuity; refraction; defocus curves; non-dependency on spectacles; patient satisfaction; perceptions of photic phenomena; and challenges related to vision when performing various daily activities.

## 2. Materials and Methods

This observational prospective study included patients who had received bilateral extended monofocal Evolux™ IOL implants following cataract extraction through phacoemulsification with the intent to achieve emmetropia. The study analyzed data from patients aged 40 years or older seeking independence from spectacles at any distance, with no active eye diseases other than cataracts and without severe dry eye. Additionally, the inclusion criteria encompassed uncomplicated cataract surgery, the absence of pupillary abnormalities, and the ability to read and understand questionnaires. Participants were excluded if they had irregular corneal astigmatism, extremely small or large pupil sizes (photopic values under 3.00 mm and/or scotopic values over 7.00 mm), a history of corneal or intraocular refractive surgery, corneal anomalies, a dislocated IOL, posterior capsule opacification, corneal astigmatism exceeding 1.00 diopter (D), or any posterior pole disease. The study identified the first 16 consecutive patients meeting these criteria who had agreed to complete questionnaires on visual outcomes after undergoing bilateral Evolux™ IOL implantation.

Regarding ethical issues, this research followed the principles of the Declaration of Helsinki and was approved by the Ethics Committee of the Hospital Arruzafa under the code 01EVOLUX.

### 2.1. Intraocular Lens

All patients underwent implantation of the Evolux™ IOL manufactured by SIFI (Aci Sant’Antonio, Catania, Italy). This IOL incorporates an advanced geometric design that enhances light distribution to improve intermediate and near vision while preserving excellent distance vision. The Evolux™ IOL facilitates a seamless transition between different focal lengths, thereby reducing photic phenomena such as halos and glare [12].

The lens is crafted from hydrophobic acrylic material with a refractive index of 1.48 and an Abbe number of 50, ensuring exceptional optical clarity and minimal light scattering. Additionally, the IOL features a negative spherical aberration of −0.27 μm, enhancing depth of focus and providing heightened visual acuity at varying distances.

The lens design incorporates a C-Loop platform to guarantee stable and centered fixation within the capsular sac, thereby reducing the risk of postoperative tilting. The Evolux™ IOL is available in a power range from +5.00 to +30.00 diopters, with increments of 1.00 D up to +10.00 D, and increments of 0.50 D from +10.5 D to +30.00 D, enabling precise customization of vision correction for each patient [12].

### 2.2. Clinical Procedure

Prior to surgery, all patients underwent a thorough ophthalmological evaluation, which included optical biometry and anterior surface tomography to determine the appropriate intraocular lens (IOL) power. Axial length, anterior chamber depth, and corneal and lens thickness were measured using the IOLMaster 700 (Carl Zeiss Meditech, Jena, Germany). The biometric data obtained were utilized to calculate the IOL power using the Barrett Universal II formula. In all cases, the selected IOL power aimed to achieve myopic values close to zero, without imposing any limitations related to axial length.

### 2.3. Surgical Procedure

Surgical procedures involving IOL implantation were carried out with an average interval of 7 days between each eye. All patients underwent cataract surgery using phacoemulsification, following standard clinical procedures. All surgeries were carried out by a single experienced surgeon using local anesthesia and a 2.2 mm microincision.

### 2.4. Postoperative Evaluation

Following IOL implantation, patients underwent regular postoperative follow-up visits to assess their visual and ocular status. Best corrected visual acuity (BCVA) and uncorrected visual acuity (UVA) were evaluated for far, intermediate, and near distances at a consistent follow-up period of 3 months post-surgery for all patients. BCVA and UVA assessments were conducted monocularly and binocularly at distances of 4 m (far), 66 cm (intermediate), and 40 cm (near). Postoperatively, a best-corrected defocus curve was performed to assess far distance vision using an ETDRS vision chart backlit at 85 cd/m^2^. Additionally, logarithmic visual acuity (VA) charts calibrated for testing at 40 cm (for near) and 66 cm (for intermediate) were used to evaluate those distances, with size increments of 0.1 logarithmic units.

Three months after surgery, defocus curves were obtained from all participants under photopic conditions. The measurements were taken binocularly, ranging from +2.00 to −4.00 D in 0.50 D steps. VA was measured using the logMAR scale, and the ETDRS optotype was utilized at a distance of 4 m. All participants’ far distance vision was evaluated with their best correction to adjust for any residual refractive errors.

The defocus tolerance was determined from the defocus curves, based on subjective assessments. Establishing a VA criterion is crucial, with 0.1 logMAR being a commonly used threshold. An absolute criterion was applied to determine the range of tolerance to blurring, based on the vergences (in diopters) that yielded VA values of 0.1 logMAR or lower.

### 2.5. Questionnaires

Patients were requested to fill out two questionnaires at a consistent follow-up period of 3 months post-surgery: the Spanish version of the Catquest-9SF patient outcomes questionnaire and the Quality of Vision (QoV) questionnaire. The Catquest-9SF is a 9-item questionnaire intended to assess limitations in daily activities due to visual impairments and satisfaction with vision after cataract surgery. It has undergone extensive validation and has been shown to be sensitive in detecting vision-related changes in quality of life. Each item on the Catquest-9SF offers 5 answer options, ranging from “no, very dissatisfied” to “yes, very satisfied”. Items A and C1–C7 focus on the difficulty patients experience in various daily activities, while Item B focuses on their satisfaction with their vision. This structure enables a detailed assessment of how vision impacts the patient’s daily life and overall well-being [13].

The QoV assesses the patient’s perception of their visual quality of life following cataract surgery. This questionnaire encompasses various key elements, including the necessity for spectacles or contact lenses, satisfaction with vision at different distances (far, intermediate, and near), and the presence of visual symptoms such as halos, starbursts, and glare. QoV items are rated with response options ranging from “never” to “very often” for the frequency of symptoms, and from “not at all” to “severe” for the severity of these symptoms. This approach allows for a comprehensive evaluation of visual functionality and patient-perceived side effects, offering a comprehensive understanding of how surgery and implantation of the Evolux™ IOL impact the patient’s daily life [14].

Both questionnaires were administered at the 3-month postoperative follow-up visit to gather data on the patient’s experience and satisfaction with their vision, and to identify any residual visual issues that could impact their quality of life.

### 2.6. Statistical Analysis

The postoperative visual results were analyzed using Python’s pandas library. The visual parameters examined included UVA for far distance vision (UDVA), intermediate vision (UIVA), and near vision (UNVA), as well as BCVA for these same distances (DCVA, DCIVA, and DCNVA, respectively) for both monocular and binocular vision.

For each visual parameter, the minimum, maximum, mean, and standard deviation (SD) values were calculated to provide an overview of the data spread. Additionally, the median and interquartile range (IQR) were calculated to provide a more detailed understanding of the data distribution. Descriptive statistics were summarized for all visual parameters under both monocular and binocular conditions.

The percentages of patients satisfied with their vision in different conditions were calculated, considering those who reported being very satisfied or completely satisfied (values ≤ 2 on a 5-point scale). Correlation analyses were performed using Spearman’s coefficient to assess the relationship between visual acuity and satisfaction or frequency of spectacle use. A *p*-value less than 0.05 was considered statistically significant.

## 3. Results

In total, 32 eyes underwent cataract surgery and were implanted with the Evolux™ IOL for this study. The participants had a mean age of 72.7 years (SD: 6.9; median: 73.0; range: 60 to 85 years), with the sample consisting of 4 men (22.2%) and 14 women (77.8%). The descriptive statistics of the baseline values can be found in Table 1.

### 3.1. Postoperative Refraction

**Figure 1:**
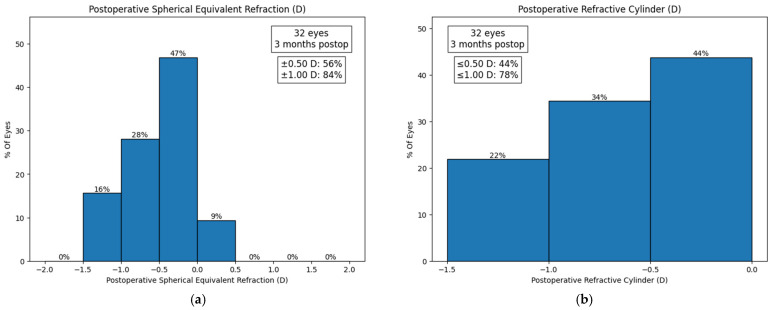
Postoperative refractive and visual results. (**a**) Postoperative spherical equivalent; (**b**) postoperative refractive cylinder. Each graph shows the percentage of eyes within different refraction ranges at 3 months postoperatively.

**Figure 2:**
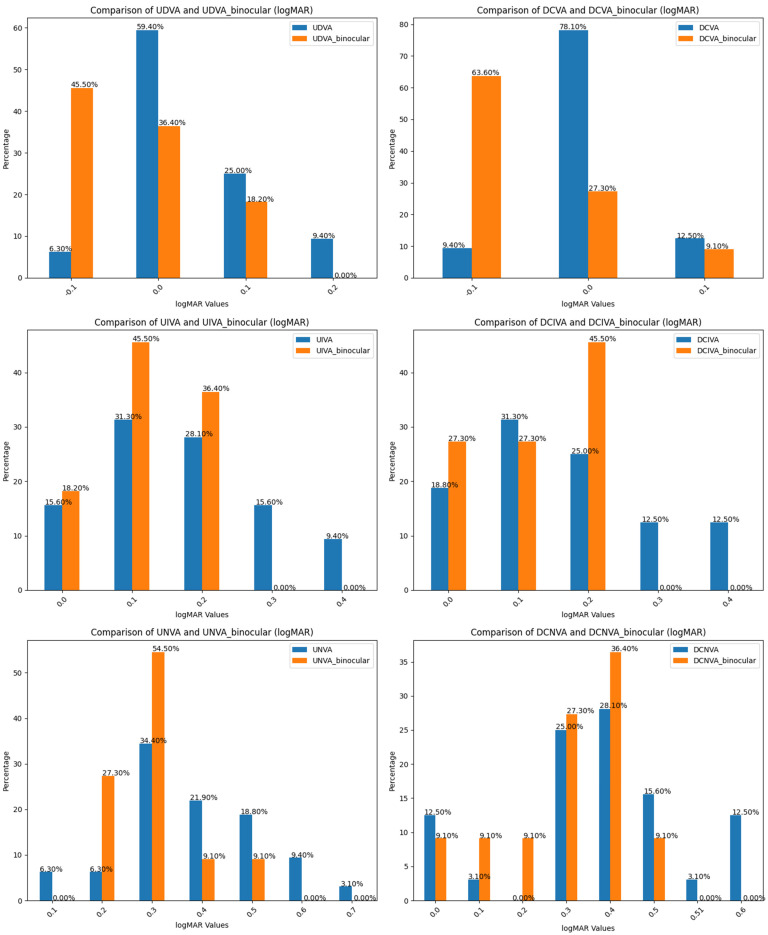
Comparison of monocular and binocular percentages of postoperative visual acuity.

In the postoperative follow-up, two patients dropped out from follow-up, leaving a total of two for the analysis of the results. The mean SE was −0.20 ± 0.39 D at 3 months postoperatively, with a median of −0.10 D and an IQR of 0.37 D. The mean residual cylinder was −0.66 ± 0.39 D at 3 months postoperatively, with a median of −0.67 D and an IQR of 0.40 D (Figure 1). Moreover, 54.5%, 93.8%, 87.5%, 87.5%, 43.8%, and 63.6% of patients achieved a UDVA, DCVA, UIVA, DCIVA, UNVA, and binocular DCNVA of 0.2 LogMAR or better (Figure 2).

Table 2 shows the VA achieved for far, intermediate, and near distances at 3 months post-surgery. The LogMAR binocular means for UDVA, UIVA, and UNVA were −0.04 ± 0.08, 0.15 ± 0.08, and 0.35 ± 0.14, respectively. The medians were −0.10, 0.20, and 0.30, with IQRs of 0.10, 0.11, and 0.29, respectively.

### 3.2. Visual Performance

**Figure 3:**
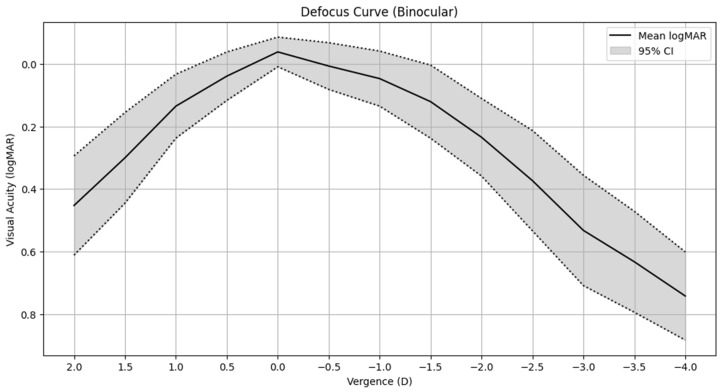
Binocular defocus curve 3 months after surgery.

Figure 3 shows the mean binocular defocus curve measured 3 months post-surgery. DCVA was better than 0.2 logMAR for defocus levels ranging from +1.00 to −3.00 D. Regarding refractive predictability, 38% and 76% of the eyes showed a postoperative SE within ±0.50 D and ±1.00 D, respectively. The mean visual performance across the defocus curve did not fall below 0.1 logMAR between +1.00 D to −2.75 D, resulting in an absolute mean defocus tolerance of 3.64 ± 0.70 D.

### 3.3. Questionnaire Results

For the Catquest-9SF questionnaire, the results were as follows: 93.8% of respondents reported not wearing spectacles for distance vision, 81.3% reported not wearing spectacles for intermediate vision, only 18.8% wore spectacles constantly for near vision, and 6.3% reported wearing spectacles for general vision. Regarding satisfaction without spectacles or contact lenses, 93.4% of patients were very satisfied or completely satisfied with their distance vision, 86.7% were satisfied with their intermediate vision, 66.7% were very satisfied or completely satisfied with their near vision, and 40.0% were completely satisfied with their overall vision without spectacles. For satisfaction in performing specific tasks, 53.3% of patients were very satisfied or completely satisfied with their ability to read the menu in a dimly lit restaurant, 86.6% were satisfied with seeing objects and reading street signs at dusk or night, 93.3% were satisfied with seeing steps or curbs in the same condition, 73.4% were very satisfied or completely satisfied with their ability to read or view photos on a smartphone or tablet, and 90.9% were satisfied with their ability to read numbers and gauges from the car’s dashboard. Detailed descriptive statistics for each question of the questionnaire can be found in Table 3.

Our analysis revealed the following significant correlations between VA and satisfaction or frequency of spectacle use: A moderate positive correlation (rho = 0.635) exists between UDVA and satisfaction without spectacles at far distance, with a highly significant *p*-value (*p* = 0.001); we also observed a moderate negative correlation (rho = −0.412) between UDVA and the frequency of use of spectacles for medium distance, with a statistically significant *p*-value (*p* = 0.041). Furthermore, a moderate positive correlation (rho = 0.635) was identified between UDVA and satisfaction without spectacles for medium distance, also with a highly significant *p*-value (*p* = 0.001). Finally, a moderate negative correlation (rho = −0.607) was found between binocular DCNVA and the frequency of spectacle use for medium distances, with a statistically significant *p*-value (*p* = 0.047). No significant correlations were found for near vision (*p* < 0.05).

**Figure 4:**
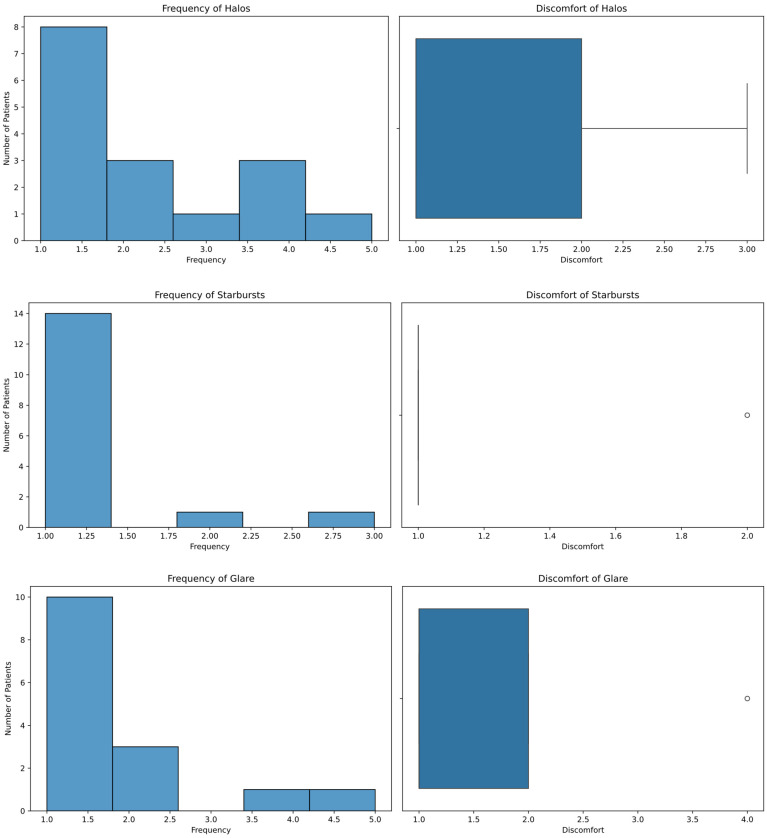
Frequency, distribution, and discomfort self-reported in the QoV questionnaire.

Patients completed the QoV questionnaire to report their experiences with visual symptoms. The mean frequency of halos reported by patients was 2.12 ± 1.41 (measured on a scale from 0 to 3), with 50% of them not experiencing halos in the past seven days, while 18.75% experienced halos rarely and another 18.75% often. The mean discomfort associated with halos was 1.50 ± 0.73 (measured on a scale from 0 to 3), with 62.5% of patients reporting no discomfort and 25% reporting mild discomfort. For starbursts, the mean frequency was 1.19 ± 0.54, and 87.50% of patients did not experience starbursts. The discomfort caused by starbursts averaged 1.06 ± 0.25, with 93.75% of patients reporting no discomfort. Regarding glare related to scattered light, the mean frequency was 1.67 ± 1.23 (measured on a scale from 0 to 3), with 66.67% of patients not experiencing glare, while 20% experienced it rarely. The discomfort caused by glare had a mean value of 1.47 ± 0.83 (measured on a scale from 0 to 3), with 66.67% of patients reporting no discomfort and 26.67% reporting mild discomfort. These results demonstrate the variability in the experience and perception of these symptoms among patients. Histograms and box plots of the frequency and discomfort for each visual symptom are shown in Figure 4.

## 4. Discussion

In our research, the Evolux™ IOL demonstrated significant efficacy in enhancing VA across various distances post-cataract surgery. Patients exhibited exceptional VA and a marked reduction in dependence on spectacles, with a minimal incidence of photic phenomena such as halos and glare. Overall satisfaction was notably high, affirming the Evolux™ IOL’s efficacy in improving postoperative quality of life. Notably, the study revealed excellent UDVA values, averaging −0.04 logMAR, comparable with findings in other enhanced monofocal IOLs evaluated in previous studies. For instance, Unsal et al. [15] observed that Tecnis Eyhance IOLs provided visual results like standard monofocal intraocular lenses but with a substantial improvement in intermediate vision. Similarly, Sihmar et al. [1] found that the Tecnis Eyhance IOL demonstrated comparable UDVA values to extended depth of focus (EDOF) IOLs such as the Tecnis Symfony, with a mean UDVA of 0.0625 logMAR at three months postoperatively, aligning with our results. A similar trend was observed for the Vivinex Impress XY1-EM IOL, which, despite having limited clinical studies, maintained excellent distance visual acuity comparable with other improved single-vision lenses [16]. Regarding ISOPURE IOLs, Ansari et al. [17] reported excellent distance vision, with 100% of patients achieving a UDVA of at least 0.1 LogMAR, reinforcing that both the Evolux™ IOL and the ISOPURE IOL offer excellent distance vision quality, positioning themselves as reliable options for improving distance vision without additional correction. Recently, Spagnuolo et al. [18], in a comparative study on the performance of the Evolux IOL versus the Eyhance IOL, found that although both lenses offered good intermediate and distance vision, the Evolux IOL demonstrated superior visual outcomes compared with the Eyhance lens. Lee et al. [19] concluded that UDVA was similar in both groups (Tecnis Eyhance IOL and EDOF IOL) at the three-month follow-up, consistent with our results. Additionally, Corbelli et al. [20] also noted excellent distance VA in groups when comparing IOLs, including Tecnis ZCB00, Eyhance, and Symfony, consistent with our findings. Similarly, Schimid et al. [21] reported excellent distance VA with the Eyhance and RayOne EMV IOLs, confirming that these extended monofocal IOLs, like the Evolux™ IOLs, provide clear and sharp vision at a distance without compromising visual performance. Collectively, these results suggest that the Evolux™ IOL, along with other extended monofocal IOLs, can offer similar visual performance to EDOF IOLs. However, further research is needed to directly compare the incidence of unwanted optical phenomena such as halos and glare.

Our findings demonstrate a significant enhancement in intermediate visual acuity with the Evolux™ IOL, surpassing standard monofocal IOLs, with an average value of 0.15 logMAR. This underscores the effectiveness of the Evolux™ IOL in improving intermediate vision, which is crucial for patients’ daily functionality. Unsal et al. [15] reported that the Tecnis Eyhance IOL offered superior intermediate visual acuity compared with standard monofocal IOLs, with an 84% rate of independence from spectacles for intermediate vision. Achieving freedom from spectacles is a primary goal in cataract surgery, and our results confirm that the Evolux™ IOL effectively achieves this, granting patients greater freedom in their daily activities without the need for additional vision correction in 81.3% of patients. Similarly, Sihmar et al. [1] found that the UIVA was significantly better in the EDOF IOL and Tecnis Eyhance IOL groups compared with traditional monofocal IOLs. These findings are consistent with ours, as the Evolux™ IOL also demonstrated an notable ability to improve intermediate vision, contributing to higher patient satisfaction. In the case of the Isopure IOL, Ansari et al. [17] found that 81% of cases achieved a UIVA of at least 20/25 at distances of 80 cm, and 50% of patients achieved this at 66 cm. The defocus curve indicated a depth of focus of 1.50 D, and there was minimal incidence of photic phenomena, similar to the findings in our study with the Evolux™ IOL. Pieh et al. [22] pointed out that the Vivinex Impress IOL had a defocus profile similar to that of the Isopure IOL, indicating good functional vision at intermediate distances. Given these similarities, it can be inferred that the Evolux™ IOL, which shares characteristics with the Isopure and Vivinex IOLs, may also provide clear vision at various distances while maintaining a low incidence of unwanted optical phenomena. This makes it a potentially beneficial option for patients requiring high visual acuity at intermediate distances. Mencucci et al. [16] confirmed that the Tecnis Eyhance IOL yielded better results for uncorrected intermediate visual acuity (UIVA) and corrected intermediate visual acuity (CIVA) compared with standard single-vision lenses. This aligns with our findings on the effectiveness of the Evolux™ IOL, suggesting that it can similarly enhance intermediate vision performance. Beltraminelli et al. [10] also observed that enhanced monofocal IOLs offered superior performance in intermediate vision at 66 cm, aligning with our results obtained with the Evolux IOL™. These findings were also confirmed by Gigon et al. [23], who compared the Tecnis Eyhance IOL with the Tecnis ZCB00 monofocal IOL and noted a marked improvement in intermediate vision in patients with the Eyhance IOL. Our results with the Evolux™ IOL further support this trend, demonstrating a significant enhancement in intermediate vision compared with traditional monofocal intraocular lenses.

Regarding near vision at 40 cm, our results showed a UNVA of 0.35 logMAR, indicating a moderate reduction in the use of glasses for close distances. Although this improvement is significant for many patients, it does not reach the levels of close correction seen with EDOF IOLs. It should be noted that 44% of eyes achieved a postoperative spherical equivalent between −1.50 and −0.50 D, which could have enhanced near visual performance. Similarly, Unsal et al. [15] found that the Tecnis Eyhance IOL did not provide remarkable improvements in near vision versus standard monofocal IOLs. This finding is consistent with our results, suggesting that the Evolux™ IOL, while effective for intermediate vision, might not be the most suitable option for patients who prioritize close tasks. Similarly, the Vivinex Impress IOL has also shown limitations in improving near vision, aligning with the results seen with the Evolux IOL™. On the other hand, Sihmar et al. [1] and Kohnen et al. [24] reported that EDOF IOLs, which have been specifically designed to extend the depth of focus, produced better near vision results than enhanced monofocal outcomes. This contrast with our results reinforces the idea that the Evolux™ IOL, like other extended monofocal lenses, is more inclined to offer advantages in intermediate and distance vision. On the other hand, targeting slight myopia (micro-monovision) could enhance near visual performance without significantly compromising distance vision, although this hypothesis requires further investigation to be validated.

Concerning the defocus curve, our postoperative results indicated that patients maintained a visual acuity better than 0.2 logMAR for defocus levels between 1.00 D and −1.50 D, indicating excellent defocus tolerance and the Evolux™ IOL’s ability to sustain good visual quality across a wide range of distances. This finding aligns with the observations of Sihmar et al. [1], who also noted that the Tecnis Eyhance IOL provided adequate visual stability over a broad spectrum of defocus. The favorable defocus tolerance observed in our study underscores the suitability of the Evolux™ IOL for individuals engaging in daily activities that demand quick changes between focusing distances, such as reading or using electronic devices. Beltraminelli et al. [22] also highlighted that extended monofocal lenses offer superior performance in intermediate vision and improved depth of focus, which is consistent with our findings regarding the ability of the Evolux™ IOL to offer functional vision over a wider range of defocus.

In terms of postoperative refraction, the average SE in our study was −0.20 ± 0.39 D, which is comparable with the results of other studies on enhanced monofocal lenses. Beltraminelli et al. [22] observed that patients with lenses tended to have slight postoperative myopia compared with those who received standard monofocal IOLs, attributing this phenomenon to the geometric design of the anterior surface of the lens. This trend towards slightly greater myopia is also consistent with our results and could be explained by the focus on optimizing intermediate vision, which could influence the distance refractive results. Mencucci et al. [16] also reported greater variability in the refractive values of the Tecnis Eyhance IOL group, suggesting that the lens design, looking to improve intermediate vision, may generate more diverse refractive results. This variability, which was also observed in our study, reflects the complexity of balancing intermediate and distance vision in enhanced monofocal IOLs. Similarly, Unsal et al. [15] and Park et al. [25] found a wider distribution of spherical values in their cohorts with Tecnis Eyhance IOLs, which reinforces the consistency of our results with the existing literature, highlighting the effectiveness of the Evolux™ IOL in achieving good refractive results, although with a slight tendency towards myopia.

Our results were also highly positive concerning patient satisfaction, with 93.8% of patients reporting not wearing spectacles for distance and 81.3% for intermediate vision. This is consistent with the studies made by Unsal et al. [15], who reported an 84% spectacles independence rate for intermediate vision with the Tecnis Eyhance IOL. Beltraminelli et al. [20] also reported high satisfaction among patients with enhanced monofocal IOLs, which was also observed in our study with the Evolux™ IOL. In addition, Mencucci et al. [16] found that improved monofocal IOLs, such as the Tecnis Eyhance IOL, offered high levels of satisfaction, especially with intermediate vision, and low spectacle use.

Regarding photic phenomena such as halos and glare, our results showed a low incidence of these side effects, which is a positive indicator for the Evolux™ IOL. This finding is consistent with those of Beltraminelli et al. [22], who observed that the Tecnis Eyhance IOL maintained good visual quality without compromising contrast sensitivity, and with a low incidence of photic phenomena. Similarly, Sihmar et al. [1] also reported a low incidence of these effects in the Tecnis Eyhance IOL group.

Our study has several strengths. First, it provides a comprehensive analysis of both objective visual results and patient-reported satisfaction, offering a holistic view of the IOL’s performance. Second, the use of validated questionnaires such as the Catquest-9SF ensures the reliable measurement of patient-reported outcomes. Finally, the comparison with other studies highlights the relative performance of the Evolux™ IOL, placing our findings within a broader context. However, there are also some limitations. The relatively small sample size limits the generalizability of findings and the robustness of the statistical analysis. In addition, the three-month follow-up period may not capture any long-term results or complications that could arise over time. Finally, the study did not include measurements in low-light conditions, which could provide additional insights into the IOL’s performance in real-world environments. Future studies should include larger sample sizes and longer follow-up periods to validate these findings and assess the long-term stability and impact of the Evolux™ IOL. In addition, incorporating objective visual results in mesopic conditions would provide a more complete understanding of the IOL’s performance under various lighting conditions. Directly comparing the Evolux™ IOL with other EDOF IOLs in randomized clinical investigations would further strengthen the evidence base and guide clinical decision-making.

## 5. Conclusions

Evolux™ IOLs have shown to be a promising solution for patients undergoing cataract surgery, offering excellent visual acuity at far, intermediate, and near distances. The results of the study indicate that these lenses provide high patient satisfaction due to the reduction in spectacle dependency and the low incidence of photic phenomena such as halos and glare. The findings suggest that Evolux™ IOLs can significantly improve postoperative quality of life. All these findings should be confirmed in a larger prospective and randomized clinical investigation.

## Figures and Tables

**Table 1:** Descriptive statistics for the baseline visit.

Parameter	Min	Max	Mean ± SD	Median (IQR)
Preoperative SE (D)	−7.50	4.25	0.19 ± 2.58	0.25 (3.12)
Preoperative J0 (D)	−1.00	0.94	−0.18 ± 0.38	−0.23 (0.32)
Preoperative J45 (D)	−0.74	0.38	−0.06 ± 0.27	−0.07 (0.38)
K1 (D)	40.71	47.34	43.88 ± 1.69	44.08 (2.44)
K2 (D)	41.42	47.74	44.48 ± 1.68	44.63 (2.49)
H (mm)	21.74	25.19	23.09 ± 0.89	22.8 (1.05)
ACD (mm)	2.03	3.66	2.98 ± 0.39	2.92 (0.53)
IOL power (D)	16.00	24.00	21.25 ± 2.14	21.50 (2.25)

SE, spherical equivalent; ACD, anterior chamber depth; K1, flat corneal meridian; K2, steep corneal meridian; IOL, intraocular lens; SD, standard deviation; IQR, interquartile range.

**Table 2:** Descriptive statistics for postoperative logMAR visual acuity.

	Min	Max	Mean	SD	Median	IQR
UDVA	−0.10	0.20	0.04	0.08	0.00	0.10
UDVA (bino)	−0.10	0.10	−0.04	0.08	−0.10	0.10
DCVA	−0.10	0.10	0.01	0.06	0.00	0.00
DCVA (bino)	−0.10	0.10	−0.06	0.06	−0.10	0.10
UIVA	0.00	0.40	0.18	0.11	0.20	0.11
UIVA (bino)	0.00	0.20	0.15	0.08	0.20	0.11
DCIVA	0.00	0.40	0.18	0.12	0.20	0.20
DCIVA (bino)	0.00	0.20	0.15	0.08	0.20	0.11
UNVA	0.20	0.60	0.37	0.11	0.30	0.20
UNVA (bino)	0.10	0.50	0.35	0.14	0.30	0.29
DCNVA	0.00	0.60	0.36	0.16	0.30	0.20
DCNVA (bino)	0.00	0.50	0.30	0.15	0.30	0.15

UDVA, uncorrected distance visual acuity; DCVA, distance-corrected visual acuity; UIVA, uncorrected intermediate visual acuity; DCIVA, distance-corrected intermediate visual acuity; UNVA, uncorrected near vision acuity; DCNVA, distance-corrected near visual acuity; bino, binocular; Min, minimum; Max, maximum; SD, standard deviation; IQR, interquartile range.

**Table 3:** Descriptive analysis of the results of the CatQuest-9SF questionnaire.

Question	Min	Max	Mean ± SD	Median (IQR)
**1. Within the last 7 days, how often did you wear glasses (including reading glasses) or CL?**				
A. To see at a far distance (1.5 m or more)	2	5	4.81 ± 0.75	5.0 (0.0)
B. To see at an intermediate distance (0.5–1.5 m)	2	5	4.56 ± 1.03	5.0 (0.0)
C. To see at a near distance (less than 0.5 m)	1	5	2.88 ± 1.41	3.0 (2.0)
D. To see in general (all distances)	2	5	4.50 ± 0.82	5.0 (1.0)
**2. What is your level of satisfaction without glasses (including reading glasses) or CL?**				
A. To see at a far distance (1.5 m or more)	1	5	1.50 ± 1.03	1.0 (1.0)
B. To see at an intermediate distance (0.5–1.5 m)	1	5	1.62 ± 1.09	1.0 (1.0)
C. To see at a near distance (less than 0.5 m)	1	5	2.25 ± 1.13	2.0 (1.3)
D. To see in general (all distances)	1	5	1.75 ± 1.00	2.0 (1.0)
**3. Generally, what is your level of satisfaction when performing these tasks?**				
A. Reading a menu in a restaurant with dimmed light	1	5	2.50 ± 1.32	2.0 (1.3)
B. Seeing objects or reading signs on the streets at sunset or night	1	5	1.94 ± 1.12	2.0 (1.0)
C. Seeing steps or borders at sunset or night	1	3	1.50 ± 0.63	1.0 (1.0)
D. Looking at photos on a smartphone or tablet	1	5	2.12 ± 1.15	2.0 (1.3)
E. Reading numbers and indicators on the car dashboard	1	3	1.36 ± 0.67	1.0 (0.5)

## Data Availability

The data presented in this study are not openly accessible due to privacy and ethical considerations; however, they can be obtained from the corresponding author upon a duly justified request.
